# Physician prescribing of opioid agonist treatments in provincial correctional facilities in Ontario, Canada: A survey

**DOI:** 10.1371/journal.pone.0192431

**Published:** 2018-02-15

**Authors:** Fiona G. Kouyoumdjian, Alexandra Patel, Matthew J. To, Lori Kiefer, Leonora Regenstreif

**Affiliations:** 1 Department of Family Medicine, McMaster University, Hamilton, Canada; 2 Centre for Urban Health Solutions, St. Michael’s Hospital, Toronto, Canada; 3 University of Toronto, Toronto, Canada; 4 Faculty of Medicine, Dalhousie University, Halifax, Canada; 5 Ontario Ministry of Community Safety and Correctional Services, Toronto, Canada; 6 Dalla Lana School of Public Health, University of Toronto, Toronto, Canada; The University of York, UNITED KINGDOM

## Abstract

**Background:**

Substance use and substance use disorders are common in people who experience detention or incarceration in Canada, and opioid agonist treatment (OAT) may reduce the harms associated with substance use disorders. We aimed to define current physician practice in provincial correctional facilities in Ontario with respect to prescribing OAT and to identify potential barriers and facilitators to prescribing OAT.

**Methods:**

We invited all physicians practicing in the 26 provincial correctional facilities for adults in Ontario to participate in an online survey.

**Results:**

Twenty-seven physicians participated, with representation from most correctional facilities in Ontario. Of participating physicians, 52% reported prescribing methadone and 48% reported prescribing buprenorphine/naloxone to patients in provincial correctional facilities. Nineteen percent of participants reported initiating methadone treatment and 11% reported initiating buprenorphine/naloxone for patients in custody. Participants identified multiple barriers to initiating OAT in provincial correctional facilities including concerns about medication diversion and safety, concerns about initiating treatment in patients who are not currently using opioids, lack of linkage with community-based providers and the Ministry of Community Safety and Correctional Services policy. Identified facilitators to initiating OAT were support from institutional health care staff and administrative staff, adequate resources for program delivery and access to linkage with community-based OAT providers.

**Conclusions:**

This study identifies opportunities to improve OAT programs and to improve access to OAT for persons in provincial correctional facilities in Ontario.

## Introduction

Research in Canada has consistently identified high rates of substance use disorders among people in jails and prisons [1–13]. The majority of persons report recent drug use at the time of admission to custody [2, 10, 14–18], including use of opioids [19–22], and many people continue to use drugs in custody [15, 16, 23–29]. There is evidence that people who experience incarceration commonly engage in behaviours such as injecting drugs [2, 3, 14, 18, 23, 24, 27, 29–38], sharing needles and other paraphernalia [3, 21, 24, 29, 33, 36, 39], and polysubstance use [2, 10, 40], which increase the risk of adverse sequelae such as overdose or infection with HIV or hepatitis C. Further, evidence from Ontario reveals that the risk of death from overdose is high in this population compared to the general population, in particular at the time of release [41, 42].

Incarceration presents a unique opportunity to offer prevention and treatment to people who use substances and who may otherwise be hard to reach [43]. Access to opioid agonist treatment (OAT) is an important means to reduce harms associated with opioid use, and has been shown to positively impact treatment retention, illicit drug use, drug-related HIV risk behaviours, criminal activity, and mortality in persons with opioid use disorder in the general population [44–48]. A recent systematic review found low quality evidence that agonist treatments were not effective in reducing drug use or criminal activity in prisoners, with pooled risk ratios for agonist pharmacological compared to no intervention of 0.72 (95% CI 0.51, 1.00) for objective drug use, 0.61 (95% CI 0.31, 1.18) for a dichotomous measure of self-reported drug use, -0.62 (95% CI -0.85, -0.39) for a continuous measure of self-reported drug use, 0.60 (95% CI 0.32, 1.14) for a dichotomous measure of arrests, 0.77 (95% CI 0.36, 1.64) for a dichotomous measure of re-incarceration, and -74.21 (95% CI -133.53, -14.89) for a continuous measure of criminal activity [49]. However, the study authors noted that the literature regarding the effectiveness of pharmacological treatment of opioid dependence in prisoners is limited in quality and quantity, which makes it challenging to determine whether the findings regarding effectiveness are valid [49]. Recent observational studies found that OAT was associated with a reduced risk of death in persons after release from prison; two studies found absolute risk differences between those exposed and unexposed of 27.4 and 30.3 deaths, respectively, per 1,000 person years in the four weeks after release [50, 51]. In this context, the World Health Organization has recommended that all prisoners with opioid use disorders should have access to methadone or other agonist treatment [52].

Further, in 1990 the United Nations articulated the principle that correctional authorities should provide access to care in correctional facilities that is at least equivalent to the care available in the community: “Prisoners shall have access to the health services available in the country without discrimination on the grounds of their legal situation”[53]. While there remain barriers to OAT access in Ontario [54], OAT is widely accessible for the treatment of opioid use disorders, and therefore persons in provincial correctional facilities should have access to OAT in custody on the basis of this principle.

There are many challenges to providing evidence-based treatments in correctional facilities, and identifying specific barriers and facilitators to the implementation of evidence-based treatments may illuminate opportunities to advance access to care and standards of care [55]. Since physicians play a central role in providing access to OAT in correctional facilities as well as in the community, we undertook a study of physician prescribing of these medications in provincial correctional facilities in Ontario. Our objective was to define current physician practice in provincial correctional facilities in Ontario with respect to prescribing OAT and to identify potential barriers and facilitators to prescribing OAT.

## Methods

### Study population

Our study population was all physicians who provide health care services in provincial correctional facilities for adults in Ontario, which is estimated at about 100 physicians [56]. Provincial correctional facilities house persons who are detained prior to sentencing and persons who are sentenced to less than 2 years, as well as persons sentenced to two years or longer prior to being transferred to a federal prison and those in temporary detention for other reasons [57].

### Recruitment

We recruited participants in two ways. First, staff in the Ministry of Community Safety and Correctional Services distributed the letter of information and survey information to Health Care Managers in each of the 26 provincial correctional facilities for adults and asked each Health Care Manager to distribute the survey information to all physicians working in the facility. Second, the investigators distributed information about the survey to eligible physicians in their professional networks. An invitation to participate was sent in November 2016 and a reminder was sent after 2 weeks.

### Survey

We developed the survey based on our experiences in correctional and community settings and based on published studies examining these factors in US prison systems [58] and other contexts [59–61]. One investigator created an initial draft and the draft was revised by other investigators for face validity, clarity and brevity. We asked participants questions about their medical specialty, in which correctional facility or facilities they worked, knowledge of the Ministry of Community Safety and Correctional Services’ policies regarding OAT, prescribing practices for methadone and buprenorphine/ naloxone and barriers and facilitators of initiating OAT (see full survey in S1). Depending on participant responses, the survey was between 13 and 15 questions. We made the survey available through an online survey tool, SurveyMonkey.

In the survey, we used the term “opioid substitution therapies” or OST in several places to describe methadone and buprenorphine/naloxone (see S1). However, in this manuscript, we use the term opioid agonist treatment or OAT to describe these treatments for opioid use disorders (except when directly quoting survey questions or participants’ comments), in order to accurately represent the nature of these treatments and to avoid perpetuating inaccurate ideas regarding the goals and impacts of these treatments [62].

### Analysis

We calculated the proportion of physicians who prescribed methadone and buprenorphine/naloxone. We examined the proportion of physicians who prescribed any methadone and any buprenorphine/naloxone, respectively, and who reported that they initiated methadone and buprenorphine/naloxone treatments for patients in custody. We examined the proportion of physicians who reported each barrier and facilitator to initiating methadone and buprenorphine/naloxone, respectively.

We compiled comments from open-ended questions and grouped them by topic through an iterative process. Specifically, we reviewed comments and grouped them into categories and then subcategories when indicated. We included all responses in either summary statements, such as the number of people who expressed a certain opinion, or by including the specific comment.

### Ethics approval

The study was approved by the Hamilton Integrated Research Ethics Board. We specified in the information and consent form that consent was implied through completion and submission of the survey.

## Results

### Participants

Twenty-seven physicians completed the survey. Participants reported working in 15 of the 26 correctional facilities for adults, including 10 of the 13 facilities with a cross-sectional population of over 200. Four participants did not specify in which institutions they worked.

Of the 27 respondents, 17 were Family Physicians, seven were Psychiatrists and three indicated that they had another specialty.

Eighteen participants (67%) reported that they knew about the Ministry of Community Safety and Correctional Services’ written health care policies on Methadone Maintenance Therapy and Buprenorphine/Naloxone Therapy. All physicians who reported prescribing buprenorphine/naloxone and 13 of the 14 physicians who prescribed methadone said they knew about the policies.

### Prescribing practices

All participants indicated that there was at least one physician who prescribed OAT in the facility or facilities where they worked.

Of all participants, 52% reported prescribing methadone and 48% buprenorphine/ naloxone for the treatment of opioid use disorders in correctional facilities (Table 1), including 12 participants (44% of all participants) who prescribed both methadone and buprenorphine/naloxone. Reasons for not prescribing methadone and buprenorphine/naloxone are shown in Table 1, with the most common reasons being not having an exemption from Health Canada to prescribe methadone (*i*.*e*. an exemption under Section 36 of the Controlled Drugs and Substances Act, which is required to prescribe methadone in Canada and for which physicians apply through the federal government or through their provincial licensing authority [63]), others being responsible for the service in the institution, not being interested in adding this treatment to current clinical work and not having adequate knowledge about the treatment.

**Table 1:** Physician prescribing of opioid agonist treatments for opioid use disorders in provincial correctional facilities for adults in Ontario, Canada, N = 27.

Survey question		n/N (%)
Do you prescribe methadone?	Yes	14/27 (52)
	No	13/27 (48)
Reasons for not prescribing methadone*	I have no exemption to prescribe methadone.	9/13 (69)
	Others are responsible for this service in my institution.	8/13 (62)
	I am not interested in adding this to my current clinical work.	7/13 (54)
	I do not have adequate knowledge about these treatments.	5/13 (38)
	I do not have enough time to add this to my current clinical work.	2/13 (15)
	I don’t think these are effective/beneficial treatments.	1/13 (8)
Do you prescribe buprenorphine/naloxone?	Yes	13/27 (48)
	No	14/27 (52)
Reasons for not prescribing buprenorphine/naloxone*	Others are responsible for this service in my institution.	10/14 (71)
	I do not have adequate knowledge about these treatments.	5/14 (36)
	I am not interested in adding this to my current clinical work.	4/14 (29)
	I do not have enough time to add this to my current clinical work.	3/14 (21)
	I don’t think these are effective/beneficial treatments.	1/14 (7)

*Percentages do not sum to 100% because categories are not mutually exclusive.

Of the 14 physicians who reported prescribing methadone in custody, all 14 (100%) reported that they maintained treatment that was initiated in the community and five (36%) reported that they also initiated patients on treatment in the correctional facility. Of the 13 physicians who reported prescribing buprenorphine/naloxone, 10 (77%) reported that they maintained treatment that was initiated in the community and three (23%) reported that they initiated treatment in the correctional facility. Of the six participants who reported initiating methadone or buprenorphine for the treatment of opioid use disorders for patients in custody, five reported initiating treatment only in specific clinical situations beyond standard criteria such as opioid use disorder and having follow up in place on release. The specific clinical situations were women with an upcoming release to the community and stable social status for methadone or buprenorphine/naloxone initiation; persons with a sentence length over one month and a positive urine drug screen and no history of not following up in the community post-release for methadone initiation; persons in opioid withdrawal with recent active use for more than two weeks and a history of injection of opioids in the past month for buprenorphine/naloxone initiation; persons who are “high risk” and have recent use of methadone for methadone initiation; and persons who no longer have withdrawal symptoms and have a reasonable chance of achieving a therapeutic dose before release for methadone initiation.

### Barriers and facilitators to initiating OAT in correctional facilities

**Fig 1:**
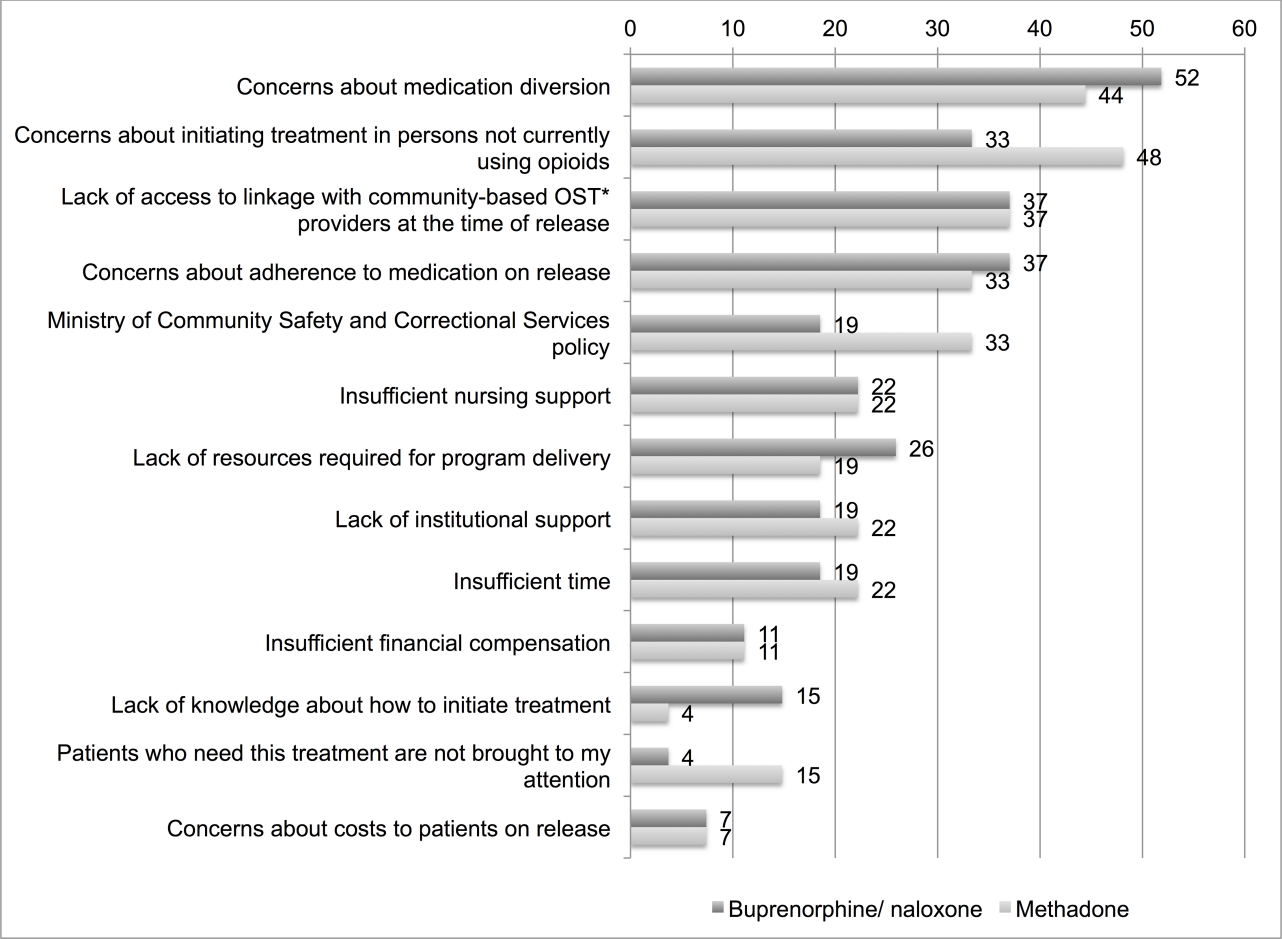
Barriers to initiating opioid agonist treatments reported by physicians working in provincial correctional facilities for adults in Ontario, Canada, % of N = 27. *OST = opioid substitution therapy, which includes opioid agonist treatments methadone and buprenorphine/naloxone.

Physicians were asked whether any of a list of factors was a barrier to initiating methadone or buprenorphine/naloxone in the correctional facility, and the results are shown (Fig 1). The most commonly identified barriers were concerns about medication diversion, concerns regarding the appropriateness of treatment initiation in custody of people who are not currently using opioids, lack of linkage with community-based OAT providers at the time of release, concerns about adherence to medication on release and the Ministry of Community Safety and Correctional Services policy.

**Fig 2:**
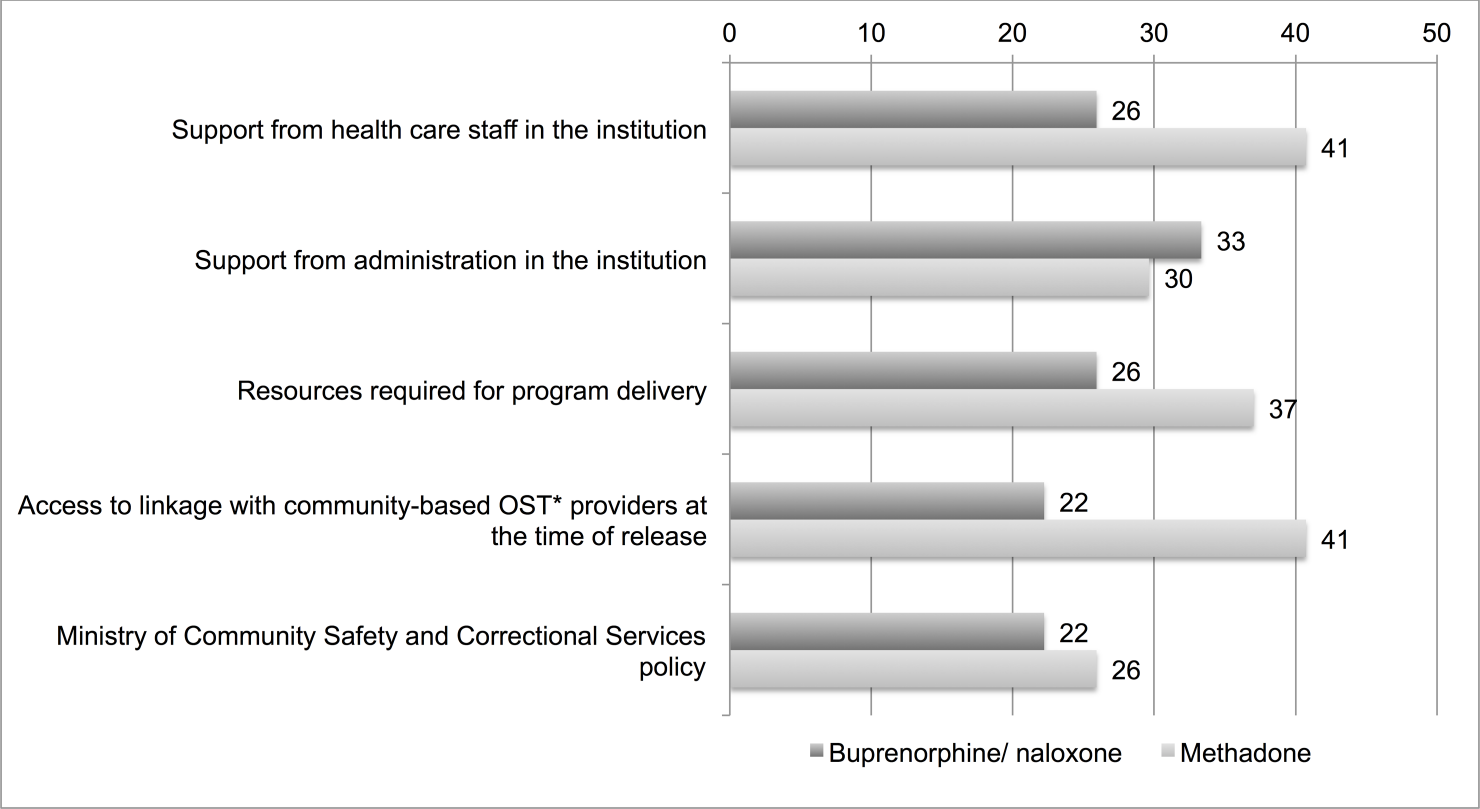
Facilitators of initiating opioid agonist treatments reported by physicians working in provincial correctional facilities for adults in Ontario, Canada, % of N = 27. *OST = opioid substitution therapy, which includes opioid agonist treatments methadone and buprenorphine/naloxone.

Over 30% of participants agreed that each of the following factors would facilitate the initiation of OAT (Fig 2): support from health care staff in the institution, support from administration in the facility, resources required for program delivery and access to linkage with community-based OAT providers at the time of release.

### Participant comments regarding OAT in correctional facilities

#### Institutional factors in OAT delivery

Three participants commented on the institutional set-up of OAT delivery. One physician wrote, “Another provider does this alone. I am excluded from this domain of assessment and prescribing.” Another participant noted that community-based physicians manage OAT: “…no local prescribing doctor… for the most part, [community-based OAT providers] have been providing the service within our jail…” A third participant reported having developed relationships with community prescribers to support continuity of care for patients on release: “Community methadone prescribers have been contacted and are aware of methadone starts… and have agreed to take patients in their programs upon release with no gap in treatment”.

Two participants identified that a single institutional staffperson may facilitate access to treatment, for example noting the importance of the “[f]requent and eloquent pleading from [a physician] to help patients with OST” and that the “Health Care Manager… is extremely helpful.” Two other participants commented on the need for collaboration, stating: “…as a Psychiatrist, I would require the help of the GP serving the jail and the requirements (or at least the way the institution interprets them) are a [deterrent]” and: “There is a huge need for psychiatry to work WITH the OST MD”.

Participants suggested ways to improve OAT delivery, such as “[a] better physical set up so the patients can be adequately monitored whilst [buprenorphine/naloxone] dissolves” and having these treatments “…handled by a designated staff physician with special interest.” Another participant wrote: “[I s]uggest policies support [physicians] making the decision and assessing patients in a manner which is not as exhaustive as current Policy, but appropriate to the patient's situation…” Two participants noted that better remuneration of physicians would facilitate OAT prescribing; one of them stated, “The time and liability with this population requires better remuneration…” Several participants identified that these treatments are “under-resourced” and impose a burden on health care staff, and one person suggested that initiation of OAT should only occur if there were appropriate resources in place: “A decision to openly allow initiating substitution therapy while incarcerated should only be initiated if the Ministry is prepared to provide appropriate support resources *i*.*e*. nursing, social services, administrative”.

#### Challenges to continuity of care

Participants noted challenges to continuity of care in custody and on release, as illustrated by this quote: “Most of the population I treat is transiently in custody. Their treatment is frequently passed from one physician to another from day to day, both because of the nature of day to day physician coverage, and inmate movement from one unit to another unit. The inmates I treat are mostly in REMAND, i.e. not yet sentenced, and therefore really any discussion about initiating MMT is out the question when there is the possibility they may not return from the next court appearance.” Two other physicians noted challenges regarding follow up on release, stating “…it would be difficult finding community physicians for patients to follow up with, and difficult to ensure that patients actually followed up,” and “[f]ollowup for this group is limited to 1 or 2 MMT programs in the community since these patients often have difficulty finding/keeping willing Primary Care.” Another participant identified other individual-level barriers: “[n]o health card on release, change of residence on release (no access to methadone clinic)…” One physician stated that follow up on release may affect access to treatment on re-admission: “If inmates fail to follow-up in the community, then upon readmission… methadone management will not be started again…”.

#### Physician attitudes about OAT effectiveness, appropriateness and safety

Several physicians questioned the appropriateness and effectiveness of OAT in this context. One wrote, “I believe it would be completely inappropriate for me to initiate [methadone in this correctional facility] even if it were within Ministry policy” and another commented that methadone and buprenorphine/naloxone were “not [an] effective way of treating opioid abuse.” Another participant stated that initiating methadone in custody was not more effective than providing counselling and referring to treatment on release. A participant commented that this treatment didn’t seem to help patients: “Because we treat an increasingly heavy burden of Methadone users, most of whom are repeat offenders, we feel overburdened, and don't feel very optimistic about how much [methadone treatment] is really helping most of this population, especially when a large percentage continue to use their [opioids] anyway. I guess we don't see the harm reduction.” Another participant commented, “Initiation of opioid substitution therapy while incarcerated should be approached with extreme caution. Inmates will quite quickly determine that it is their right, and our obligation that we provide this service.” A participant suggested that OAT should be weaned instead of maintained: “We do not initiate [OAT…]. The program is not being used for its true purpose. [This facility] does wean methadone and suboxone unfortunately outside agencies do not. [I]t is a program that is meant to be [w]eaned and observed.” In contrast, two physicians expressed support for the provision of OAT in custody: “I think [OAT] is …misunderstood as a treatment modality that is potentially helpful to many inmates.” “Initiation of methadone/ suboxone…is beneficial to the health and addiction concerns of inmates if appropriate follow-up in the community upon release is completed”.

Three participants noted the lack and potential value of other types of treatment in custody, in addition to or independent from OAT: “Shouldn't all patients on methadone/ suboxone be required to attend substance use disorder counseling/ educational/ relapse prevention groups?” “I would be happy to have much stronger abstinence programs and counseling.” “I often see methadone used as a substitute for proper treatment counselling and abstinence”.

Many participants expressed concern regarding the safety and diversion of these medications. One physician wrote, “I have initiated [these treatments in custody] a few times, but every time the methadone or suboxone has been misused or diverted. Therefore, I generally do not initiate methadone or suboxone now. I am concerned there would be a significant problem with misuse if initiating opioid maintenance therapy in the institutions became common practice.” Another participant commented that “… [m]ethadone and suboxone could easily become used as "currency" between inmates, and there would be major safety implications regarding this, and a major risk of overdose” and “continuing opioid maintenance therapy is appropriate, but initiating it is a very slippery slope and I would have major safety concerns we would see a high level of diversion and overdose.” Regarding methadone in particular, a participant noted the need for “[s]afeguards to ensure medication is not diverted”.

Two participants identified specific risks of diversion with buprenorphine/naloxone: “…The current formulation ([sublingual] tabs) make this product easily divertable…” and “Despite extra and resource depleting measures to ensure proper ingestion, there have been far more attempts of diversion with Suboxone than with [m]ethadone.” Another participant suggested the need to advocate for the dissolvable form of buprenorphine/ naloxone: “We need to use BRAND Suboxone, not the generic, within institutions because the generic is much easier to hoard/divert. Better yet, we should partner with appropriate [government] and pharma to prompt the decision making bodies to approve the version of suboxone in the form of a dissolvable strip for use in Canada…”.

Three physicians commented on patients’ frequent concurrent use of and seeking of other prescribed and non-prescribed medications, with comments such as, “Many still use cocaine yet will still get methadone,” “Inmates…seek lyrica, gabapentin, stimulants, quetiapine, hs sedation, benzodiazepines with factitious complaints,” and “[Another physician in the institution] prescribes lots of [benzodiazepines] which is dangerous given concurrent use of OST, the ubiquity of illicit drugs in jail, and the trouble with detecting ODs. [O]versedated inmates are quiet and aren't easily detected as they suffocate…quietly to death”.

## Discussion

This study of physicians working in provincial correctional facilities identified that about half of participants prescribed methadone and half prescribed buprenorphine/naloxone. The most common reasons for physicians not prescribing these treatments were not having an exemption to prescribe methadone, other physicians being responsible for the service in the institution, not being interested in adding this treatment to clinical work and not having adequate knowledge. About one third of those prescribing methadone and almost one quarter of those prescribing buprenorphine/naloxone reported initiating patients on treatment, often only in specific circumstances beyond standard medical indications. Across participants, commonly identified barriers to the initiation of OAT in custody were concerns about medication diversion and safety, concerns about initiating these treatments in patients who are not currently using opioids, challenges to continuity of care including linkage with community-based providers and the Ministry of Community Safety and Correctional Services policy. Identified facilitators of OAT initiation included support from institutional health care and administrative staff, adequate resources required for program delivery and access to linkage with community-based OAT providers.

While no published research in Canada has explored these issues, our findings are consistent with limited research from the US regarding access to OAT in prisons and institutional staff attitudes. A representative sample of correctional agencies in the USA in 2004 and 2005 found that 0.9% of prisons and 54.5% of jails had a methadone maintenance program [64]. A 2008 national survey of medical directors of state prison systems found that only 55% of prison systems in the US provided methadone to prisoners in any circumstance and only 14% provided buprenorphine [58]. The most common reasons specified for not offering OAT in prisons were that the facility favoured drug-free detox, security concerns and costs [58]. Limited partnerships with community providers was one of the most common reasons why referrals were not offered on release, as well as the facility preferring drug-free detox and the facility focusing on inmate health during incarceration [58]. A 2006 survey of medical staff and case managers in the Connecticut Department of Corrections found that most participants agreed that OAT is “just substituting one addiction for another” and thought that the goal of OAT “should always be eventual detoxification and sobriety,” while a majority did not agree that methadone services should be expanded so that “those who used narcotics before incarceration who want opiate substitution therapy can receive it” [65]. A phone survey was conducted in 2014 and 2015 with administrators in city jails, county jails and prisons that house women in the USA, with participation of facilities in 40 states, and found that 21.7% of facilities (10/53) reported providing methadone maintenance therapy for women with opioid addiction during pregnancy [66].

As noted, the equivalence principle and scientific evidence support the provision of OAT in correctional facilities [44–48, 50, 51, 53]. Further, both the College of Physicians and Surgeons of Ontario and the Methadone Treatment and Services Advisory Committee to the Ontario Ministry of Health and Long-Term Care recommend access to OAT for persons in correctional facilities in Ontario [54, 67]. Yet physicians’ responses in this survey suggest that people in provincial correctional facilities in Ontario may face substantial barriers to initiating treatment with OAT, due to a variety of issues at the provider and structural levels.

There are potential limitations to the study. First, of the estimated 100 physicians practicing in provincial correctional facilities in Ontario [56], only 27 participated. We do not know whether the study information was shared with all physicians. There was participation from physicians who were working in most facilities and including most of the larger facilities. We do not have access to a list of physicians who work in provincial correctional facilities or to their demographic or practice characteristics, so we are unable to compare those who did and did not participate. There may be systematic bias in participation based on prescribing practices or attitudes regarding OAT, and it is not possible to determine whether and how this would affect results. Second, while it would be valuable to understand experiences with OAT from the perspectives of other health care staff, administrative staff and patients, we only included physicians given our interest in interventions to address gaps between knowledge and action for physicians [68] and for feasibility reasons. Finally, since we did not ask about whether any physician in the correctional facility initiated treatment with methadone or buprenorphine/naloxone, we can not determine whether patients have access to these treatments in each correctional facility.

These results suggest opportunities to improve OAT programs in custody and to improve access to OAT for persons in custody. Targeted continuing medical education opportunities could support physician prescribing of OAT and the provision of high quality care. To address challenges to continuity of care, correctional facilities could establish and develop linkages with community-based programs. As data emerge on the effectiveness of different programs and OAT formulations, best practices should be defined and implemented to prevent OAT misuse and diversion. This may require advocacy for safer formulations such as dissolvable buprenorphine/naloxone, as well as ensuring adequate resources for program implementation.

Future research should explicitly consider other perspectives with respect to access to OAT in custody. Quantifying individual and population-level benefits and risks associated with these treatments in custody would also be valuable. With increasing opioid-related mortality in the US and Canada due to high potency opioids such as fentanyl and its analogues [69, 70], OAT has the potential to have an even greater impact on outcomes such as death and nonfatal overdose in people in custody and at the time of release. Finally, any initiatives to improve access to care and to influence physician prescribing practices should be evaluated to understand impacts on processes, patient outcomes and the satisfaction of patients, physicians, other health care providers and institutional staff.

## Conclusions

This survey of physicians working in provincial correctional facilities in Ontario, Canada revealed that about half of participants prescribed methadone and buprenorphine/ naloxone and only a minority of participants initiated patients on these treatments in custody. Further work can be done to improve access to OAT and to improve the organization and delivery of OAT programs in provincial correctional facilities. The health care and correctional systems should collaborate to close gaps in care for the benefit of persons in custody and to improve population health.

## Supporting information

S1 File Survey. (DOCX)
