# Supplementary material for: Physician prescribing of opioid agonist treatments in provincial correctional facilities in Ontario, Canada: A survey
Source: PLoS One. 2018 Feb 15;13(2):e0192431. doi: 10.1371/journal.pone.0192431 (PMC5813939; doi:10.1371/journal.pone.0192431)
Supplement: S1 File — (DOCX) [file pone.0192431.s001.docx]

**S1 File.** **Survey**

1. What is your medical specialty?

☐ Family Medicine

☐ Psychiatry

☐ Other (please specify):

2. In which provincial correctional facility or facilities do you work?

3. How many physicians prescribe methadone or buprenorphine/naloxone (Suboxone) for the treatment of opioid use disorders in the facility in which you work? (If you work in more than 1 facility, please specify the number in each facility)

4. Do you know about the Ministry of Community Safety and Correctional Services written health care policies on Methadone Maintenance Therapy and Buprenorphone/Naloxone Therapy?

☐ Yes

☐ No

*Methadone prescribing*

5. Do you prescribe methadone for the treatment of opioid use disorders in provincial correctional facilities?

☐ Yes

☐ No

If no to #5:

6. Why not? (check all that apply):

☐ I have no exemption to prescribe methadone.

☐ I am not interested in adding this to my current clinical work.

☐ I do not have enough time to add this to my current clinical work.

☐ I do not have adequate knowledge about these treatments.

☐ Others are responsible for this service in my institution.

☐ I don’t think these are effective/beneficial treatments.

☐ Other (please specifiy):

If yes to #5:

6a. When do you prescribe methadone for the treatment of opioid use disorders in the correctional facility where you work (check all):

☐ Maintaining treatment

☐ Initiating treatment

6b. If you initiate treatment with methadone for the treatment of opioid use disorders in patients in custody, in which patients do you initiate treatment? (list clinical criteria such as age, gender, comorbidities, drug use characteristics such as recent injection or overdose; situational criteria such as whether sentenced and/or length of sentence, whether has an existing relationships with community-based OST provider, etc.)

☐ I don’t initiate treatment with methadone for patients in custody

☐ Other (please specify):

*Buprenorphine/naloxone (Suboxone) prescribing*

7. Do you prescribe *buprenorphine/naloxone (Suboxone)* for the treatment of opioid use disorders in provincial correctional facilities?

☐ Yes

☐ No

If no to #7:

8. Why not? (check all that apply):

☐ I am not interested in adding this to my current clinical work.

☐ I do not have enough time to add this to my current clinical work.

☐ I do not have adequate knowledge about these treatments.

☐ Others are responsible for this service in my institution.

☐ I don’t think these are effective/beneficial treatments.

☐ Other (please specify):

If yes to #7:

8a. When do you prescribe buprenorphine/naloxone (Suboxone) for the treatment of opioid use disorders in the correctional facility where you work (check all):

☐ Maintaining treatment

☐ Initiating treatment

8b. If you initiate treatment with buprenorphine/naloxone (Suboxone) for the treatment of opioid use disorders in patients in custody, in which patients do you initiate treatment? (list clinical criteria such as age, gender, comorbidities, drug use characteristics such as recent injection or overdose; situational criteria such as whether sentenced and/or length of sentence, whether has an existing relationships with community-based OST provider, etc.)

☐ I don’t initiate treatment with buprenorphine/naloxone (Suboxone) for patients in custody.

☐ Other (please specify):

*Barriers and facilitators to initiation of opioid substitution therapy*

9. What are barriers to initiating treatment with methadone in the correctional facility where you work?

☐ Ministry of Community Safety and Correctional Services policy

☐ Lack of institutional support

☐ Insufficient nursing support

☐ Insufficient financial compensation

☐ Insufficient time

☐ Lack of knowledge about how to initiate

☐ Lack of resources required for program delivery

☐ Concerns about diversion of medication

☐ Concerns about the appropriateness of initiation of treatment in custody in persons who are not currently using opioids

☐ Patients who need this treatment are not brought to my attention

☐ Concerns about costs to patients on release

☐ Concerns about adherence to medications on release

☐ Lack of access to linkage with community-based OST providers at the time of release

☐ Other (please specify):

10. What are facilitators of initiating treatment with methadone in the correctional facility?

☐ Ministry of Community Safety and Correctional Services policy

☐ Support from administration in the institution

☐ Support from health care staff in your institution

☐ Resources required for program delivery

☐ Access to linkage with community-based OST providers at the time of release

☐ Other (please specify):

11. What are barriers to initiating treatment with buprenorphine/naloxone (Suboxone) in the correctional facility where you work?

☐ Ministry of Community Safety and Correctional Services policy

☐ Lack of institutional support

☐ Insufficient nursing support

☐ Insufficient financial compensation

☐ Insufficient time

☐ Lack of knowledge about how to initiate

☐ Lack of resources required for program delivery

☐ Concerns about diversion of medication

☐ Concerns about the appropriateness of initiation of treatment in custody in persons who are not currently using opioids

☐ Patients who need this treatment are not brought to my attention

☐ Concerns about costs to patients on release

☐ Concerns about adherence to medications on release

☐ Lack of access to linkage with community-based OST providers at the time of release

☐ Other (please specify):

1. What are facilitators of initiating treatment with buprenorphine/naloxone (Suboxone) in the correctional facility where you work?

☐ Ministry of Community Safety and Correctional Services policy

☐ Support from administration in the institution

☐ Support from health care staff in your institution

☐ Resources required for program delivery

☐ Access to linkage with community-based OST providers at the time of release

☐ Other (please specify):

13. Do you have any other comments regarding prescribing opioid substitution therapies in provincial correctional facilities?

Thank you for completing this survey.
